# *Sporothrix schenckii sensu stricto* and *Sporothrix brasiliensis* Are Differentially Recognized by Human Peripheral Blood Mononuclear Cells

**DOI:** 10.3389/fmicb.2017.00843

**Published:** 2017-05-10

**Authors:** José A. Martínez-Álvarez, Luis A. Pérez-García, Erika Mellado-Mojica, Mercedes G. López, Iván Martínez-Duncker, Leila M. Lópes-Bezerra, Héctor M. Mora-Montes

**Affiliations:** ^1^Departamento de Biología, División de Ciencias Naturales y Exactas, Universidad de GuanajuatoGuanajuato, Mexico; ^2^Centro de Investigacion y de Estudios Avanzados del Instituto Politécnico NacionalIrapuato, Mexico; ^3^Laboratorio de Glicobiología Humana y Diagnóstico Molecular, Centro de Investigación en Dinámica Celular, Instituto de Investigación en Ciencias Básicas y Aplicada, Universidad Autónoma del Estado de MorelosCuernavaca, Mexico; ^4^Laboratory of Cellular Mycology and Proteomics, Biology Institute, University of Rio de Janeiro StateRio de Janeiro, Brazil

**Keywords:** *Sporothrix schenckii*, *Sporothrix brasiliensis*, host-fungus interaction, cell wall, cytokine, mononuclear cells, sporotrichosis, innate immune sensing

## Abstract

*Sporothrix schenckii sensu stricto* and *S. brasiliensis* are usually associated to sporotrichosis, a subcutaneous mycosis worldwide distributed. Comparative analyses between these two species indicate they contain genetic and physiological differences that are likely to impact the interaction with host cells. Here, we study the composition of the cell wall from conidia, yeast-like cells and germlings of both species and found they contained the same sugar composition. The carbohydrate proportion in the *S. schenckii sensu stricto* wall was similar across the three cell morphologies, with exception in the chitin content, which was significantly different in the three morphologies. The cell wall from germlings showed lower rhamnose content and higher glucose levels than other cell morphologies. In *S. brasiliensis*, the wall sugars were constant in the three morphologies, but glucose was lower in yeast-like cells. In *S. schenckii sensu stricto* cells most of chitin and β1,3-glucan were underneath wall components, but in *S. brasiliensis* germlings, chitin was exposed at the cell surface, and β1,3-glucan was found in the outer part of the conidia wall. We also compared the ability of these cells to stimulate cytokine production by human peripheral blood mononuclear cells. The three *S. schenckii sensu stricto* morphologies stimulated increased levels of pro-inflammatory cytokines, when compared to *S. brasiliensis* cells; while the latter, with exception of conidia, stimulated higher IL-10 levels. Dectin-1 was a key receptor for cytokine production during stimulation with the three morphologies of *S. schenckii sensu stricto*, but dispensable for cytokine production stimulated by *S. brasiliensis* germlings. TLR2 and TLR4 were also involved in the sensing of *Sporothrix* cells, with a major role for the former during cytokine stimulation. Mannose receptor had a minor contribution during cytokine stimulation by *S. schenckii sensu stricto* yeast-like cells and germlings, but *S. schenckii sensu stricto* conidia and *S. brasiliensis* yeast-like cells stimulated pro-inflammatory cytokines via this receptor. In conclusion, *S. brasiliensis* and *S. schenckii sensu stricto*, have similar wall composition, which undergoes changes depending on the cell morphology. These differences in the cell wall composition, are likely to influence the contribution of immune receptors during cytokine stimulation by human monocytes.

## Introduction

Fungal infections are a significant burden to both healthy and hospitalized populations, representing about 15% of hospital-acquired infections (Brown et al., 2012). *Sporothrix schenckii sensu lato* is a cosmopolitan and dimorphic fungal pathogen, and the causative agent of human and animal sporotrichosis, an infection transmitted by contact of the subcutaneous tissue with contaminated material or infected animals (Mora-Montes et al., 2015; Zhang et al., 2015; Rodrigues et al., 2016). This fungal disease is worldwide distributed, and a significant number of cases have been reported in North and South America, Asia, some African countries and Australia (Chakrabarti et al., 2015). It is an emergent infection in immunocompromised patients, and an occupational disease in farmers and workers in close contact with soil, wood, bark, forage, and straw (Lopez-Romero et al., 2011). *S. schenckii sensu lato* is in fact a complex of at least four closely related species: *S. schenckii sensu stricto, S. brasiliensis, S. globosa*, and *S. lurei* (Rodrigues et al., 2015; de Beer et al., 2016); which have significant differences in the host range (Rodrigues et al., 2013, 2016; Mora-Montes et al., 2015), virulence (Fernandes et al., 2000, 2013; Brito et al., 2007; Arrillaga-Moncrieff et al., 2009; Fernandez-Silva et al., 2012a; Castro et al., 2013; Clavijo-Giraldo et al., 2016), and sensitivity to antifungal drugs (Marimon et al., 2008; Fernández-Silva et al., 2012b; Rodrigues et al., 2014; Borba-Santos et al., 2015). Among the complex members, *S. schenckii sensu stricto* and *S. brasiliensis* are the most common species associated to human and animal sporotrichosis, respectively (Chakrabarti et al., 2015; Mora-Montes et al., 2015).

The innate and adaptive immune responses are the main host defense mechanisms to control and eradicate fungal pathogens (Martinez-Alvarez et al., 2014). The study of the interaction between the immune system and either *Candida albicans, Aspergillus fumigatus*, or *Cryptococcus neoformans*, the most thoroughly studied models of fungal pathogenesis, has demonstrated that the cell wall, and the capsule in the case of *C. neoformans*, are the main sources of pathogen-associated molecular patterns recognized by pattern recognition receptors (PRRs) found on innate immune cells (Díaz-Jiménez et al., 2012; Latge and Beauvais, 2014; Netea et al., 2015; Leopold Wager et al., 2016). Using *C. albicans* as a model, it has been demonstrated that β1,3-glucan is sensed by dectin-1 and TLR2, and plays a major role in the induction of pro-inflammatory cytokines and phagocytosis by macrophages (Gantner et al., 2005; Gow et al., 2007; Heinsbroek et al., 2008). Mannose receptor (MR), dectin-2, and DC-SIGN participate in the *N*-linked mannan sensing, a cell wall component with a significant role in both cytokine stimulation and macrophage uptake (Netea et al., 2006; Mora-Montes et al., 2007, 2010a; Cambi et al., 2008; McKenzie et al., 2010; Saijo and Iwakura, 2011). Although recognized by TLR4, *O*-linked mannans have a minor role in the stimulation of cytokine production (Netea et al., 2006), but play a negative role during phagocytosis by macrophages (McKenzie et al., 2010). Finally, chitin has a negative impact during interaction with peripheral blood mononuclear cells (PBMCs) (Mora-Montes et al., 2011), and is a strong stimulus for IL-10 production in a MR-, TLR9-, and NOD2-dependent mechanism (Wagener et al., 2014).

Despite this significant progress, little is known about the *S. schenckii sensu lato* cell wall composition, organization, and relevance during the host-fungus interaction. Thus far, it is well established that *S. schenckii sensu lato* cell wall contains a significant number of antigenic molecules recognized by anti-*Sporothrix* antibodies (Ruiz-Baca et al., 2011, 2014); but the specific contribution of cell wall components during interaction with innate immune cells is currently unknown. Using the animal model of sporotrichosis, it has been demonstrated that TLR4 recognizes lipidic extracts from yeast cells and triggers the production of both pro- and anti-inflammatory cytokines (Sassá et al., 2009, 2012). Furthermore, TLR2 also contributes to the recognition of this organism, participating in the phagocytosis of yeast cells by macrophages, and the production of both pro- and anti-inflammatory cytokines (Negrini et al., 2013). Using human THP-1-derived macrophages, MR has been involved in the phagocytosis of *S. schenckii* conidia (Guzman-Beltran et al., 2012).

Here, to understand the relevance of the cell wall of conidia, yeast-like cells and germlings of *S. brasiliensis* and *S. schenckii sensu stricto* during the interaction with human PBMCs, we performed a comparative study of the wall composition of the different morphotypes of *S. schenckii sensu stricto* and *S. brasiliensis.*

## Materials and Methods

### Strains and Culturing Conditions

*Sporothrix schenckii* 1099-18 (ATCC MYA 4821) and *S. brasiliensis* 5110 (ATCC MYA 4823), both clinical isolates (Castro et al., 2013), were used in this study. Fungal cells were maintained and propagated at 28°C in YPD medium (1% [w/v] yeast extract, 2% [w/v] gelatin peptone, 3% [w/v] dextrose), added with 2% (w/v) agar when required. Conidia were obtained by growing the fungus on YPD, pH 4.5 plates for 6–9 days at 28°C. Then, cells were harvested by placing 5 mL of sterile Phosphate Buffered Saline (PBS) and gently scraping the plate surface with a spreader. Yeast cells were obtained by growing 1 × 10^6^ conidia/mL in 20 mL YPD, pH 7.8, and incubating 18 h at 37°C and shaking (120 rpm). An aliquot of 10 mL was then inoculated in 40 mL of YPD, pH 7.8, and incubated for 4–7 days at 37°C and shaking (120 rpm). Under these conditions, nearly 100% cells displayed a yeast-like morphology. Germlings were obtained incubating conidia for 11–12 h in YPD, pH 4.5 at 28°C and shaking (120 rpm). In all cases, cells were pelleted by centrifuging at 2700 × *g* for 10 min, and washed twice with PBS. For interaction with human cells, the fungal cell concentration was adjusted at 2 × 10^6^ cell/mL in RPMI 1640 Dutch modification (Sigma) supplemented with 2 mM L-glutamine (Sigma), 100 μM pyruvate (Sigma), and 50 μg/mL gentamicin (Sigma). Cells were immediately used for cytokine stimulation or kept at -20°C until used. To remove cell wall *O*-linked glycans, cells from the three different morphologies were β-eliminated with 0.1 M NaOH as previously reported (Diaz-Jimenez et al., 2012), washed twice with sterile PBS and cell density adjusted to 2 × 10^6^ cells/mL. Under these conditions, more than 96% cells kept viability, as tested by CFU/mL before and after treatment with NaOH. Cell heat-killing was achieved by incubating at 60°C for 2 h. Under these conditions, cells lost viability but did not burst, as measured by the amount of 280nm- and 260nm-absorbing material released into the extracellular medium upon incubation (less than 3 ± 0.5% of the total absorbance obtained upon mechanical disruption). Loss of cell viability was confirmed on YPD plates incubated at 28°C for 7 days.

### Cell Wall Analysis

Cells were washed twice with deionized water and mechanical disrupted in a Braun homogenizer, with 16 cycles of 30 s and cooling on ice in-between (Mora-Montes et al., 2010b). Cell walls were pelleted by centrifuging and cleaned with sequential incubations with hot 2% (v/v) SDS (BioRad), 0.3 M β-mercaptoethanol (Sigma), and 1 M NaCl (Sigma), as described (Mora-Montes et al., 2007). Freeze-dried cell walls were hydrolyzed by adding 2 M trifluoroacetic acid (Sigma) and boiling for 3 h; then, the acid was evaporated, and samples were suspended in deionized water. The hydrolysates were analyzed by High-Performance Anion-Exchange Chromatography coupled to Pulsed Amperometric Detection (HPAEC-PAD, Thermo Fisher Scientific) as described by Estrada-Mata et al. (2015). Protein content was quantified using alkali-hydrolyzed cell walls (Mora-Montes et al., 2007), and the colorimetric Bradford protein assay.

The relative cell wall porosity to DEAE-dextran of the three morphotypes was determined as described elsewhere (De Nobel et al., 1990). Briefly, aliquots containing 1 × 10^8^ cells were suspended in either 10 mM Tris-HCl, pH 7.4 (buffer A), buffer A plus 30 μg/mL DEAE-dextran (MW. 500 kDa, Sigma) or buffer A plus 30 μg/mL poly-L-lysine (MW. 30–70 kDa, Sigma), and incubated for 30 min at 30°C with gentle shaking at 200 rpm. Cells were pelleted by centrifuging at 11 000 × *g* for 2 min, supernatants saved and centrifuged once again before reading the absorbance at 260 nm. The relative cell wall porosity to DEAE-dextran was calculated as described by De Nobel et al. (1990).

### Analysis of Polysaccharide Exposure at the Cell Wall Surface

For chitin staining, cells were stained with 100 μg/mL fluorescein isothiocyanate-wheat germ agglutinin conjugate (WGA-FITC; Sigma) (Mora-Montes et al., 2011); whereas β1,3-glucan was labeled with 5 μg/mL IgG Fc-Dectin-1 chimera (Graham et al., 2006) for 40 min at room temperature, followed by incubating with 1 μg/mL donkey Anti-Fc IgG-FITC (Sigma) for 40 min at room temperature (Marakalala et al., 2013). Samples were inspected under fluorescence microscopy, using a Zeiss Axioscope-40 microscope and an Axiocam MRc camera (Zeiss). Pixels associated to 300 fluorescent cells were analyzed as reported (Estrada-Mata et al., 2015).

### Ethics Statement

The inclusion and use of human cells was approved by Universidad de Guanajuato (permission number 17082011 granted by Ethics Committee). Only healthy adult volunteers were enrolled in this study, and blood samples were withdrawn after information of the study was provided and the written informed consent was signed.

### Human PBMCs-*Sporothrix schenckii sensu lato* Interaction

Human PBMCs were isolated by density centrifugation using Histopaque-1077 (Sigma) as described (Endres et al., 1988). Cells were washed twice in sterile PBS and suspended in RPMI 1640 Dutch modification (Sigma). The human cell-fungus interaction was conducted in 96-well microplates, with a total volume of 200 μL containing 5 × 10^5^ human PBMCs and 1 × 10^5^ *Sporothrix* cells. In some experiments, human PBMCs were pre-incubated for 60 min at 37°C with 5% (v/v) CO_2_ with either 200 μg/mL laminarin (Sigma), 10 μg/mL anti-MR (Invitrogen, Cat. No. Mab-Hmr), 10 μg/mL anti-TLR4 (Santa Cruz Biotechnology, Cat. No. sc-293072), or 10 μg/mL anti-TLR2 (eBioscience, Cat. No. 16-9922) prior to stimulation with fungal cells. Isotype matched, irrelevant IgG_1_ antibodies (10 μg/mL, Santa Cruz Biotechnology, Cat. No. sc-52003) were used as control for experiments assessing MR and TLR4; while IgG_2_aκ (10 μg/mL, eBioscience, Cat. No. 14-4724-85) was used as control when TLR2 was analyzed. All reagents used for the study were tested negative for the presence of LPS (assessed with the *Limulus* amebocyte lysate from Sigma); nonetheless, all reactions were conducted in presence of 5 μg/mL polymyxin B (Sigma) (Schwartz et al., 1972). In all cases, plates were incubated for 24 h at 37°C with 5% (v/v) CO_2_, then centrifuged for 10 min at 1800 × *g* and 4°C, and supernatants saved and kept at -20°C until used. Tumor necrosis factor alpha (TNFα), interleukin 6 (IL-6), and interleukin 10 (IL-10) were quantified by ELISA with Standard ABTS ELISA Development kits from Peprotech. The interleukin 1β (IL-1β) production was also determined by ELISA, with a DuoSet ELISA Development kit from R&D Systems. Wells containing only human PBMCs and RPMI 1640 Dutch modification (Sigma) were included in all the interactions as control reactions (labeled as control in the corresponding figures).

### Statistical Analysis

Statistical analysis was performed using GraphPad Prism 7 software. Cytokine stimulation using human PMBCs was performed in duplicate with eight healthy donors; while other experiments were performed three times in duplicate. Data represent cumulative results of all experiments performed and are showed as mean ± SD. The Mann-Whitney *U* test was used to establish statistical significance, which was set at *P* < 0.05.

## Results

### *S. schenckii sensu stricto* and *S. brasiliensis* Have Similar Cell Wall Composition But Show Morphology-Dependent Changes

To analyze the proportions of the main cell wall components in *S. schenckii sensu stricto* and *S. brasiliensis*, cultures were set to obtain enrichment of yeast-like cells, conidia and germlings (see Materials and Methods for technical details) and cell walls from the three morphologies were isolated, acid-hydrolyzed (Mora-Montes et al., 2007, 2012), and the amount of the basic unit of chitin (N-acetylglucosamine), β-glucan (glucose), and *N*- and *O*-linked glycans (mannose, rhamnose, and glucuronic acid) (Travassos, 1989; Lopes-Alves et al., 1992; Mora-Montes et al., 2007) were quantified by HPAEC-PAD. *S. schenckii sensu stricto* and *S. brasiliensis* yeast-like cells, conidia and germlings contained the three polysaccharides analyzed (**Table 1**). Chitin content was significantly variable in the three morphologies of *S. schenckii sensu stricto*, being conidia the morphology with the lowest amount of glucosamine, and the germlings showing the highest content of this sugar (**Table 1**). No significant differences were observed in the mannose content among the three morphologies, but rhamnose was significantly lower in the germling cell wall (**Table 1**). Furthermore, glucan content was significantly different between yeast-like cells and germlings (**Table 1**). For the three morphologies in both organisms, glucuronic acid was only detected in trace amounts (not shown). Similarly, the three morphologies of *S. brasiliensis* had the analyzed sugars as part of the cell wall, with no differences in the glucosamine, mannose and rhamnose content among them (**Table 1**). Yeast-like cells contained lower glucose content than germlings (**Table 1**). When compared with the same morphology of *S. schenckii sensu stricto*, the cell wall of *S. brasiliensis* conidia had higher chitin content and lower rhamnose levels; while *S. brasiliensis* germlings contained lower chitin content and a higher amount of rhamnose (**Table 1**). No differences were observed in the protein content across the three morphologies of the two organisms studied (**Table 1**). Next, to further explore differences in the glycosylation of cell wall proteins, we assessed the wall porosity, a parameter that changes depending on the status of the protein glycosylation pathways (De Nobel et al., 1990; Estrada-Mata et al., 2015; Navarro-Arias et al., 2016; Perez-Garcia et al., 2016). The cell wall porosity of *S. schenckii sensu stricto* germlings was reduced, when compared to the porosity of conidia from the same organism, or *S. brasiliensis* germlings; while the cell wall porosity of *S. brasiliensis* conidia was significantly lower to that found in *S. schenckii sensu stricto* conidia or *S. brasiliensis* germlings (**Table 1**).

**Table 1:** Cell wall analysis of different *S. schenckii sensu stricto* and *S. brasiliensis* morphotypes.

	Cell wall abundance				Porosity (%)^∥^	Protein (μg)^¶^
Organism/Morphotype	Glucosamine (%)	Mannose (%)	Glucose (%)	Rhamnose (%)		
***S. schenckii s. str.***						
Yeast-like cells	15.7 @ 5.4	21.3 ± 7.2	39.5 @ 4.2	23.4 @ 5.4	83.4 @ 16.8	184.4 @ 23.1
Conidia	4.8 @ 0.2	22.3 ± 4.4	46.3 @ 7.5	26.6 @ 3.4	86.9 @ 2.0	180.1 @ 14.7
Germlings	25.7 @ 1.2ˆ*	16.0 ± 1.5	52.0 @ 1.4^†^	6.2 @ 1.0ˆ*	67.5 @ 1.9ˆ**,ˆ^††^	183.8 @ 11.2
***S. brasiliensis***						
Yeast-like cells	9.24 @ 0.6	27.1 ± 3.7	43.1 @ 2.3^††^	20.6 @ 1.8	74.6 @ 18.3	180.9 @ 19.7
Conidia	7.9 @ 0.6^‡^	26.2 ± 3.6	52.7 @ 7.7	13.1 @ 3.6^‡^	53.4 @ 20.5ˆ**,ˆ^††^	178.4 @ 21.5
Germlings	10.9 @ 2.2^‡^	20.0 ± 6.8	51.5 @ 2.9	17.7 @ 6.4^‡^	89.2 @ 6.2	183.5 @ 19.1

*^∥^Relative to DEAE-Dextran. ^¶^μg of protein/mg of cell wall. ^∗^*P* < 0.05, when compared to other *S. schenckii s. str.* morphologies. ^†^*P* < 0.05, when compared to *S. schenckii s. str.* yeast cells. ^‡^*P* < 0.05, when compared to the same morphology in *S. schenckii s. str.*^∗∗^*P* < 0.05, when compared to *S. schenckii s. str.* conidia. ^††^*P* < 0.05, when compared to *S. brasiliensis* germlings.*

**FIGURE 1:**
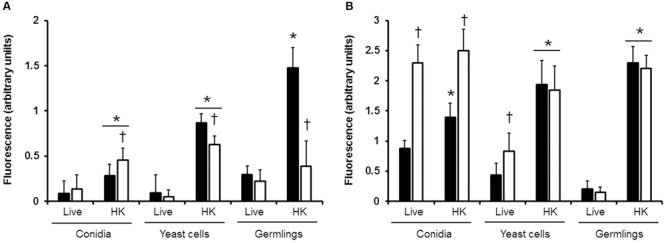
**Exposure of chitin and β1,3-glucan at the cell wall surface of different morphologies of *S. schenckii sensu stricto* and *S. brasiliensis*.** Live or heat-killed cells (HK) from *S. schenckii sensu stricto* (closed bars) or *S. brasiliensis* (open bars) were incubated with either WGA-FITC **(A)** or IgG Fc-Dectin-1and then with FITC-conjugated IgG **(B)** to label chitin or β1,3-glucan, respectively. Three hundred cells were analyzed for each group. ^∗^*P* < 0.05, when compared with the live cells; ^†^*P* < 0.05 when compared to *S. schenckii sensu stricto* cells under the same morphology and condition.

To assess whether the β1,3-glucan and chitin fibers in the cell wall of the different morphologies of *S. schenckii sensu stricto* and *S. brasiliensis* are underneath the glycoprotein layer, as reported in other fungal organisms (Steele et al., 2005; Gow et al., 2007; Mora-Montes et al., 2011; Estrada-Mata et al., 2015; Perez-Garcia et al., 2016), cells were incubated with WGA-FITC to label chitin or with the IgG Fc-Dectin-1 chimera (Graham et al., 2006), and the lectin-β1,3-glucan interaction revealed with FITC-conjugated IgG. Results indicated that chitin is equally labeled in the three morphologies of both species analyzed (**Figure 1A**). However, when cells were heat-killed (HK), a process that exposes inner wall components on the cell surface (Steele et al., 2005; Gow et al., 2007), chitin labeling was significantly higher in conidia and yeast-like cells of *S. schenckii sensu stricto* and *S. brasiliensis*, suggesting that, in live cells, most of this polysaccharide is found underneath other cell wall components (**Figure 1A**). For the case of *S. schenckii sensu stricto* germlings, a similar observation was found, but no significant differences were recorded when the fluorescence associated to live and HK *S. brasiliensis* germlings was analyzed, indicating that most of the chitin is found at the cell wall surface (**Figure 1A**). Furthermore, significant differences were observed in the ability to label HK conidia, HK yeast-like cells and HK germlings from the two species analyzed (**Figure 1A**). When a similar approach was used to determine the exposure of β1,3-glucan on the surface of the three morphologies of these *Sporothrix* species, we found that HK yeast-like cells and germlings from *S. schenckii sensu stricto* and *S. brasiliensis* displayed more labeling of this cell wall polysaccharide than the live cells, suggesting that most of the β1,3-glucan is naturally masked by components from the outer layer of the wall (**Figure 1B**). Conidia from *S. schenckii sensu stricto*, but not those from *S. brasiliensis*, followed the same trend (**Figure 1B**). When a comparison in the ability of the lectin to label cells from these two species was conducted, we found higher labeling of live and HK conidia and live yeast cells from *S. brasiliensis*. Overall, these data indicate that the *S. schenckii sensu stricto* and *S. brasiliensis* cell wall composition and organization are similar, but have morphology-dependent changes.

### *S. schenckii sensu stricto* and *S. brasiliensis* Are Differentially Recognized by Human PBMCs

**FIGURE 2:**
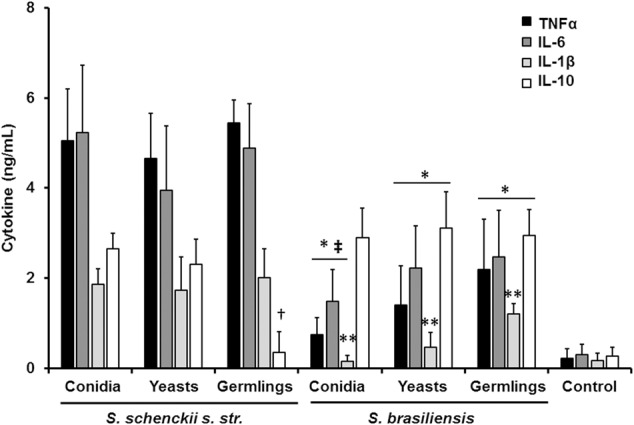
***Sporothrix schenckii sensu stricto* and *S. brasiliensis* are differentially recognized by human PBMCs.** Human PBMCs were co-incubated 24 h with live conidia, yeast-like cells or germlings from either *S. schenckii sensu stricto* (*s. str*.) or *S. brasiliensis*, the supernatant collected and used to quantify the cytokine levels. ^∗^*P* < 0.05, when compared with the cytokine level stimulated by *S. schenckii sensu stricto*; ^†^*P* < 0.05, when compared with other *S. schenckii sensu stricto* morphologies; ^‡^*P* < 0.05, when compared with *S. brasiliensis* germlings; ^∗∗^*P* < 0.05, when compared against other *S. brasiliensis* morphologies. Mock interactions of human PBMCs with culture medium were included as a control condition (control).

The differences in the cell wall composition and organization could indicate that the *S. schenckii sensu stricto* and *S. brasiliensis* sensing by innate immune cells is species specific. Thus, in order to get insights of such interaction, different morphologies of both species were co-incubated with human PBMCs and the level of secreted pro- and anti-inflammatory cytokines was quantified. Live conidia, yeast-like cells and germlings from *S. schenckii sensu stricto* stimulated similar levels of TNFα, IL-6, and IL-1β (**Figure 2**). Interestingly, for the case of the anti-inflammatory mediator IL-10, germlings stimulated lower levels of this cytokine (**Figure 2**). Results from similar experiments using different *S. brasiliensis* morphologies showed that the three morphologies of this species stimulated lower levels of TNFα and IL-6, when compared to the counterparts in *S. schenckii sensu stricto* (**Figure 2**). Conidia from both species stimulated the production of similar amounts of IL-10, but *S. brasiliensis* yeast-like cells and germlings stimulated higher IL-10 production than the same morphologies in *S. schenckii sensu stricto* (**Figure 2**). Moreover, we also observed a different ability to stimulate cytokine production among the *S. brasiliensis* morphologies: conidia stimulated the production of lower amounts of TNFα and IL-6 than germlings; and production of IL-1β was dependent on the morphology, with the lowest production stimulated by *S. brasiliensis* conidia and the highest associated to germlings (**Figure 2**). Control reactions where the human cells were incubated only with culture medium yielded threshold cytokine levels (**Figure 2**). Altogether, these data clearly indicate a species-specific and morphology-dependent ability to stimulate cytokine production by human PBMCs.

### Role of Inner Cell Wall Components and *O*-Linked Glycans in the Sensing of *S. schenckii sensu stricto* and *S. brasiliensis*

**FIGURE 3:**
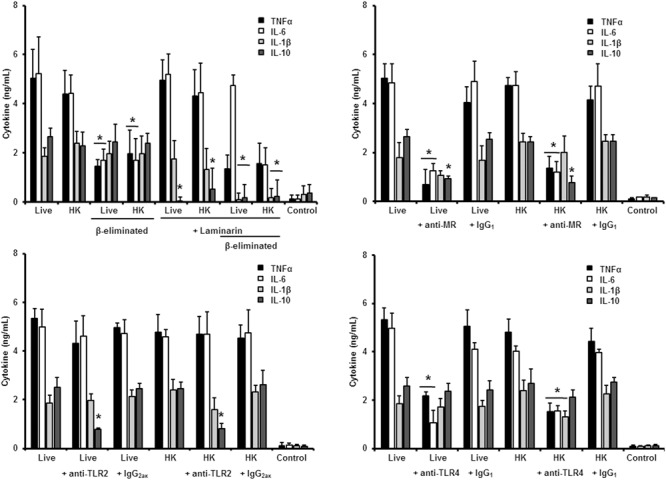
**Cytokine production by human PBMCs stimulated with *S. schenckii sensu stricto conidia*.** Human PBMCs were co-incubated 24 h with either live, HK, live and β-eliminated, or HK and β-eliminated conidia, supernatant collected and used to quantify the cytokine levels. Alternatively, human PMBCs were pre-incubated with laminarin, anti-MR, anti-TLR2 or anti-TLR4 antibodies before interaction with fungal cells. Irrelevant, isotype-matching antibodies were used as controls, as indicated. ^∗^*P* < 0.05, when compared with untreated cells.

**FIGURE 4:**
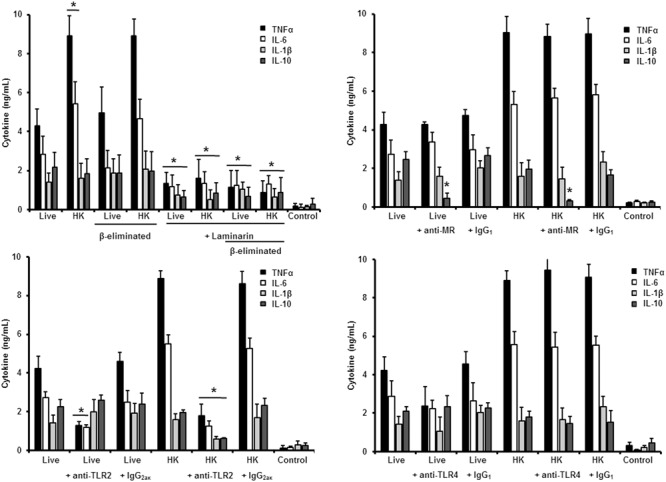
**Cytokine production by human PBMCs stimulated with *S. schenckii sensu stricto* yeast-like cells.** As **Figure 3**, but yeast-like cells were used. ^∗^*P* < 0.05, when compared with untreated cells.

**FIGURE 5:**
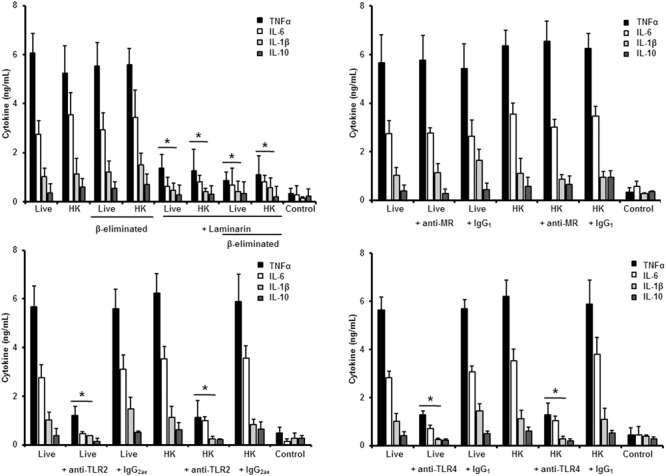
**Cytokine production by human PBMCs stimulated with *S. schenckii sensu stricto* germlings.** As **Figure 3**, but germlings were used. ^∗^*P* < 0.05, when compared with untreated cells.

**FIGURE 6:**
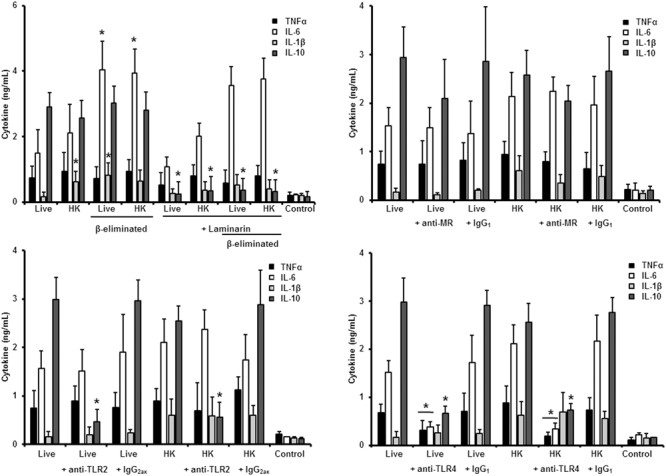
**Cytokine production by human PBMCs stimulated with *S. brasiliensis conidia*.** Human PBMCs were co-incubated 24 h with either live, HK, live and β-eliminated, or HK and β-eliminated conidia, supernatant collected and used to quantify the cytokine levels. Alternatively, human PMBCs were pre-incubated with laminarin, anti-MR, anti-TLR2, or anti-TLR4 antibodies before interaction with fungal cells. Irrelevant, isotype-matching antibodies were used as controls, as indicated. ^∗^*P* < 0.05, when compared with untreated cells.

**FIGURE 7:**
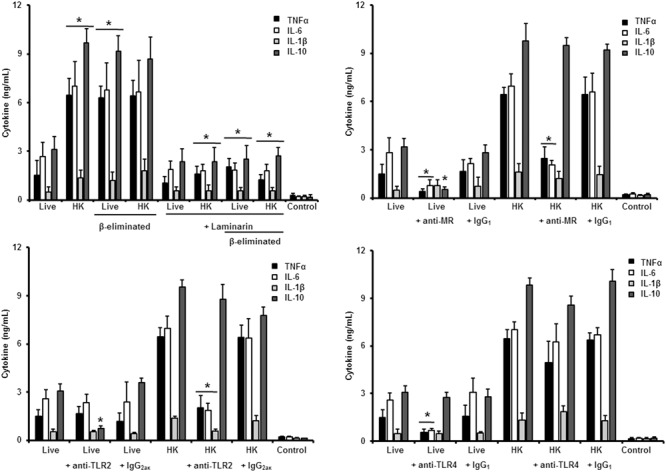
**Cytokine production by human PBMCs stimulated with *S. brasiliensis* yeast-like cells.** As **Figure 6**, but yeast-like cells were used. ^∗^*P* < 0.05, when compared with untreated cells.

**FIGURE 8:**
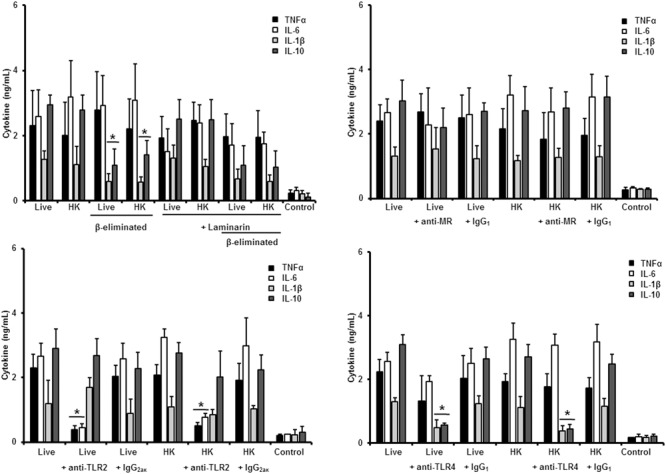
**Cytokine production by human PBMCs stimulated with *S. brasiliensis* germlings.** As **Figure 6**, but germlings cells were used. ^∗^*P* < 0.05, when compared with untreated cells.

Next, to analyze the importance of inner cell wall components during the fungus-PBMC interaction, we compared the ability of live and HK cells to stimulate cytokine production. Exposure of inner wall components did not influence the ability of conidia or germlings from *S. schenckii sensu stricto* to stimulate the production of TNFα, IL-1β, IL-6, or IL-10 (**Figures 3, 5**), but production of TNFα and IL-6 significantly increased upon interaction with HK yeast-like cells from *S. schenckii sensu stricto* (**Figure 4**). For *S. brasiliensis*, exposure of inner cell wall components at the cell surface had a different influence on cytokine production: HK conidia stimulated higher levels of IL-1β, when compared to live cells (**Figure 6**); HK yeast-like cells stimulated increased levels of TNFα, IL-1β, IL-6, and IL-10 (**Figure 7**); while live and HK germlings stimulated similar levels of cytokine production (**Figure 8**). Since *O*-linked glycans in *S. schenckii sensu lato* are remarkably different from those described in other fungal pathogens (Lopes-Alves et al., 1992; Mora-Montes et al., 2015), we next assessed the contribution of the cell wall *O*-linked glycans during the interaction with human PBMCs. This wall component was trimmed from cells by β-elimination, a treatment with minimal influence in cell viability, and previously used to characterize the contribution of this cell wall component during the host-fungus interaction (Estrada-Mata et al., 2015; Navarro-Arias et al., 2016; Perez-Garcia et al., 2016). In *S. schenckii sensu stricto*, β-eliminated conidia lost the ability to fully stimulate the production of TNFα and IL-6, indicating that they are partially required for stimulation of these cytokines (**Figure 3**). However, β-elimination did not influence the ability of live or HK yeast-like cells and germlings to stimulate cytokine production, suggesting that *O*-linked glycans are dispensable during the interaction of these morphologies with human PBMCs (**Figures 4, 5**). When a similar approach was conducted to study the different morphologies of *S. brasiliensis*, we observed that conidia had increased ability to stimulated IL-6 production, whereas live conidia, but not HK cells, also positively influenced the production of IL-1β (**Figure 6**). Similarly, upon β-elimination, live yeast-like cells stimulated higher levels of TNFα, IL-1β, IL-6, or IL-10 (**Figure 7**), suggesting that *O*-linked glycans in conidia and yeast-like cells are hidden key cell wall components for the stimulation of these cytokines. In germlings, removal of *O*-linked glycans from the cell wall negatively influenced the production of IL-1β, and IL-10, suggesting that this cell wall component is required for stimulation of these cytokines when *S. brasiliensis* germlings interact with human PBMCs. Collectively, these data indicate that inner cell wall components and *O*-linked glycans are required during the interaction of *S. schenckii sensu lato* with human PBMCs, in a species-specific and morphology-dependent manner.

### Role of PRRs in the Sensing of Different Morphologies of *S. schenckii sensu stricto*

To study the contribution of some PRRs during *S. schenckii sensu stricto* immune sensing, human PBMCs were pre-incubated with antagonists of specific PRRs before the interaction with fungal cells. To assess the importance of dectin-1 during this interaction, the human cells were pre-incubated with the specific antagonist laminarin (Maneu et al., 2011; Huang et al., 2012; Cohen-Kedar et al., 2014; Estrada-Mata et al., 2015; Navarro-Arias et al., 2016; Perez-Garcia et al., 2016); whereas anti-MR, anti-TLR4 and anti-TLR2 antibodies were used to evaluate the contribution of these receptors during the recognition of *S. schenckii sensu stricto* cells. Stimulation of TNFα and IL-6 by conidia was independent on dectin-1 recognition, even when cells were HK or β-eliminated (**Figure 3**). Production of IL-10 was strongly dependent on signaling through dectin-1, as live or HK conidia stimulated significantly lower levels of this cytokine (**Figure 3**). Loss of both, *O*-linked glycans and recognition of β1,3-glucan had a negative impact on the stimulation of IL-1β, suggesting both dectin-1 and the receptor of *O*-linked glycans work in collaboration for the stimulation of IL-1β production. The blocking of MR did not affect IL-1β stimulation, but a significant decrement in the levels of TNFα, IL-6, and IL-10 were observed upon pre-incubation with anti-MR antibodies (**Figure 3**). Loss of the signaling via TLR2 only affected the stimulation of IL-10; while preincubation with anti-TLR4 antibodies had a negative impact on the ability of live conidia to stimulate TNFα and IL-6 production. When HK cells were used, IL-1β stimulation was also reduced (**Figure 3**). Altogether, these data indicated that TNFα and IL-6 production was strongly depended on engagement of MR and TLR4 to their ligands, while IL-10 was mainly stimulated via dectin-1, TLR2, and MR. The IL-1β stimulation by *S. schenckii sensu stricto* conidia was critically depended on the recognition of β1,3 glucan and *O*-linked glycans.

When the same strategy was used to analyze the sensing of *S. schenckii sensu stricto* yeast-like cells by human PBMCs, we found that laminarin negatively influenced the stimulation of TNFα, IL-1β, IL-6, and IL-10 when live, HK or β-eliminated yeast-like cells were used in the interaction assays, suggesting a key role for dectin-1 in the sensing of this *S. schenckii sensu stricto* cell morphology (**Figure 4**). Interestingly, the production of pro-inflammatory cytokines was insensitive to the blockage of MR, but it was not the case during stimulation of IL-10 (**Figure 4**). Disruption of the signaling pathway via TLR4, did not affect the cytokine stimulation by this cell morphology; but, upon blockage of TLR2, TNFα, and IL-6 levels were significantly reduced when either live or HK cells were used in the stimulation assays (**Figure 4**). In the case of HK yeast-like cells, IL-1β and IL-10 levels were also diminished as consequence of TLR2 blocking (**Figure 4**). These data suggest that recognition of *S. schenckii sensu stricto* yeast cells by human PBMCs mainly occurs via dectin-1 and TLR2, with a modest participation of MR and TLR4.

In the case of *S. schenckii sensu stricto* germlings, our results indicate that, as in the case of yeast-like cells, pre-incubation of human PBMCs with laminarin significantly reduced the levels of TNFα, IL-1β, and IL-6 stimulated by live, HK or β-eliminated germlings (**Figure 5**). Preincubation of the immune cells with the anti-MR antibody did not affect the cytokine production stimulated by this fungal cell morphology; but blocking assays with antibodies against TLR4 or TLR2 significantly reduced the level of pro-inflammatory cytokine quantified (**Figure 5**). For all the cases, no modifications in the IL-10 levels were observed, maybe because the cytokine levels were already closer to the detection limit in the system without blocking agent (**Figure 5**).

Therefore, sensing of *S. schenckii sensu stricto* germlings by human PBMCs is mainly driven via dectin-1, TLR2 and TLR4, with a minor participation of MR. For the three morphologies, control assays, with an isotype matched, irrelevant antibody stimulated similar levels of cytokines as the system with no antibodies included (**Figures 3**–**5**).

### Role of PRRs in the Sensing of Different Morphologies of *S. brasiliensis*

We next analyzed the contribution of some PRRs during the interaction of different morphologies of *S. brasiliensis* with human PBMCs, following a similar strategy. Different from that reported for *S. schenckii sensu stricto, S. brasiliensis* conidia stimulated reduced levels of IL-10 when either live, HK or β-eliminated cells were used in the interactions with human PBMCs pre-incubated with laminarin, suggesting that engagement of dectin-1 with its ligand is part of the signaling pathway involved in the stimulation of this cytokine by *S. brasiliensis* conidia (**Figure 6**). The dectin-1 blockage did not effect the level of the pro-inflammatory cytokines analyzed (**Figure 6**). The MR was dispensable for cytokine production stimulated by this fungal cell morphology, whereas only IL-10 levels were reduced upon blocking with anti-TLR2 antibodies (**Figure 6**). Assays in presence of anti-TLR4 antibodies revealed that TNFα, IL-6, and IL-10 levels were significantly reduced upon stimulation with either live of HK conidia, with no changes in the production of IL-1β (**Figure 6**). Together, these data suggest that IL-10 production depends on interaction of dectin-1, TLR2 and TLR4 with their ligands; whereas TNFα and IL-6 stimulation is critically depended on TLR4. Surprisingly, none of the studied PRRs has an impact in the stimulation of IL-1β by *S. brasiliensis* conidia.

In the case of *S. brasiliensis* yeast-like cells, presence of laminarin during the interaction with human PBMCs did not affect the cytokine production stimulated by live yeast-like cells, but strongly reduced the levels of TNFα, IL-6, IL-1β, and IL-10 when HK cells or β-eliminated cells (in both live and HK forms) were used, indicating that in live yeast cells β1,3-glucans are not significantly exposed and that, upon the artificial exposure of this cell wall polymer at the cell surface, it is accessible to be engaged with dectin-1 (**Figure 7**). Our data showed that MR was required for proper production of TNFα and IL-6 by both live and HK yeast cells, but in the former, MR was also essential for IL-1β stimulation (**Figure 7**). The blocking of TLR2 with specific antibodies significantly reduced IL-10 stimulation by live yeast cells, but when HK cells were used, absence of the signaling via TLR2 negatively affected the production of the pro-inflammatory cytokines analyzed, but not IL-10 levels (**Figure 7**). Interestingly, pre-incubation with anti-TLR4 antibodies reduced the concentration of TNFα, IL-6, and IL-1β stimulated by live yeast-like cells. Collectively, these data suggest that β1,3-glucan is normally not accessible to be sensed by dectin-1, and that MR and TLR4 are required for stimulation of cytokine production by human PBMCs. TLR2 contributes in the signaling of IL-10 production when immune cells are interacting with live yeast-like cells.

Finally, when a similar strategy was used to study the human PBMCs-*S. brasiliensis* germlings interaction we found that pre-incubation of the immune cells with laminarin did not influence the cytokine production stimulated by live, HK or β-eliminated cells (**Figure 8**). Similarly, no significant involvement of MR in the cytokine stimulation by human PBMCs was documented (**Figure 8**). The stimulation of TNFα and IL-6 was significantly diminished upon blocking with anti-TLR4 antibodies; while IL-1β and IL-10 production was depended on the interaction of TLR4 with its ligand (**Figure 8**).

Therefore, sensing of *S. brasiliensis* germlings by human PBMCs depends on interaction of TLR4 and TLR2 with their ligands, with no major participation of dectin-1 and MR. For the three *S. brasiliensis* morphologies, the control assays, with an isotype matched, irrelevant antibody stimulated cytokines at a similar level of the system with no antibodies included (**Figures 6**–**8**).

## Discussion

Sporotrichosis has been traditionally related to the causative agent *S. schenckii*; however, in recent years, it has been thoroughly demonstrated that this species is in fact a group of phylogenetically related species named the *S. schenckii* complex (Lopez-Romero et al., 2011; Mora-Montes et al., 2015). *S. schenckii sensu stricto* and *S. brasiliensis* are, thus far, the most studied species of the complex, and several reports have demonstrated they display different phenotypical traits, as expected for two organisms that diverged about 3.8–4.9 million years ago, and with a different content in putative encoding genes (10 293 vs. 9 091, for *S. schenckii sensu stricto* and *S. brasiliensis*, respectively) (Teixeira et al., 2014). Here, we aimed to compare the cell wall composition of all morphotypes of these two species and to assess the relevance of this organelle during the interaction with innate immune cells. We decided to use primary human cells instead of cell lines, simply because the latter have defects in the programs that govern the cell biology, and sometimes do not represent the biology of non-immortalized, primary cells (Pan et al., 2009; Hannan et al., 2010). Thus, primary cells are likely to reflect closer an *in vivo* interaction than immortalized cell lines. Our data showed that the composition of the *S. schenckii sensu stricto* cell wall contrast from those reported in the eighties (Travassos and Lloyd, 1980), despite we used the same strain. However, the relative proportion of the different sugars remains constant in the three morphologies. Differences in the methodology to isolate cell walls, hydrolysis of polysaccharides, the sensitivity and the state of the art techniques applied may account for those differences. In addition, it has been reported in *S. schenckii* and other fungal species, that differences in the growth conditions are likely to have an impact on the cell wall composition (Mendonca et al., 1976; Ene et al., 2012a,b). Here, in contrast to other studies, where conversion to yeast-like cells is stimulated in brain heart infusion, we used the same culturing medium to induce the morphological transition, minimizing the influence of nutrient diversity and concentration in the analysis of the cell wall composition. In the *Candida* genus, most of the species studied contain the same wall components, but in different proportions (Estrada-Mata et al., 2015; Navarro-Arias et al., 2016; Perez-Garcia et al., 2016), and here we observed the same for *S. schenckii sensu stricto* and *S. brasiliensis*. In fungal species such as *C. albicans, A. fumigatus, Histoplasma capsulatum*, and *Paracoccidioides brasiliensis*, it has been reported that the cell wall composition depends on the morphological phase of the organism; with significant changes that have a profound impact during the host-fungus interaction (Mora-Montes et al., 2009; Lara-Lemus et al., 2014; de Oliveira et al., 2015; Lee and Sheppard, 2016); therefore, there is not an obvious reason to suspect this is not the case in *S. schenckii sensu lato*.

As reported in *C. albicans* and *A. fumigatus*, the three *S. schenckii sensu stricto* morphologies have most of the wall chitin and β1,3-glucan underneath an outer layer of wall components, most likely glycoproteins (Steele et al., 2005; Gow et al., 2007). However, this is not the case for *S. brasiliensis* cells: while yeast-like cells follow a similar trend, conidia and germlings have most of the β1,3-glucan and chitin naturally exposed at the wall surface, respectively. This is unlikely to be related to increased levels of these polysaccharides in the cell wall, as they are present in the same abundance in the three *S. brasiliensis* morphologies (**Table 1**). Since the protein, mannose, and rhamnose content is similar between *S. brasiliensis* conidia and germlings, it is tempting to speculate that glycans attached at the wall surface might be shorter, but abundant, forming a thin outer layer incapable to mask inner wall components. Alternatively, it might be possible that glycans are composed of long carbohydrate chains heterogeneously scattered on the wall surface, leaving areas where inner wall components are unprotected. Further experiments are required to provide a conclusive explanation to these observations. Nevertheless, the organization of the *S. schenckii sensu stricto* and *S. brasiliensis* cell wall is different, and is likely to affect the interaction with human PBMCs and other immune cells.

The same cytokine profile stimulated by *S. schenckii sensu stricto* conidia and yeast-like cells can be explained by the similarity in composition and polysaccharide organization between these two morphologies. Germlings stimulated lower levels of IL-10 and this might not be related to the increased content in chitin, as these cells have most of this polysaccharide hidden in the inner layer of the wall; therefore, it is possible to suggest that the lower rhamnose content in the *S. schenckii sensu stricto* cell wall might account for this observation. Indeed, it has been reported that in *Pseudallescheria boydii*, another fungal species that contains rhamnomannan at the cell wall, purified rhamnose-containing oligosaccharides are capable of inducing IL-10 production in murine macrophages (Figueiredo et al., 2010). In the case of the cytokine profile stimulated by the three morphologies of *S. brasiliensis*, we could not find a correlation between the analyzed wall components and differences in the cytokine profile elicited by the morphologies of this organism. The limitation of our study is that we did not use several isolates from the two analyzed species, and it might be possible that the differences reported here may be strain specific rather than species specific. Similar studies with other isolates from these two species are required to confirm our observations. In addition, we did not address the lipid composition, the diversity of glycosidic links found in oligosaccharides and polysaccharides, nor the diversity in the protein content. It is important to notice that previous reports on cell surface glycolipids show only the presence of mannosylinositolphosphorylceramides (Manpalpha1– > 6Ins; Manpalpha1– > 3Manpalpha1– > 6Ins; Manpalpha1– > 6Manpalpha1– > 3Manpalpha1– > 3Manpalpha1– > 6Ins; Manpalpha1– > 2Manpalpha1– > 6Manpalpha1– > 3Manpalpha1– > 3Manpalpha1– > 6Ins) (Loureiro y Penha et al., 2001). It has been previously reported that glycolipids isolated from the cellular surface have a profound impact on the interaction of *S. schenckii* with immune cells (Carlos et al., 2009), and that the diversity of cell wall proteins predicted to be in the cell wall of *S. brasiliensis* and *S. schenckii sensu stricto* is different (Teixeira et al., 2014). Thus, these and other parameters should be explored before drawing a possible explanation to the variation in the IL-1β levels stimulated by *S. brasiliensis* morphologies. It was interesting to note that stimulation of TNFα and IL-6 levels tended to be lower in the three morphologies of *S. brasiliensis* than in *S. schenckii sensu stricto*, and for yeast-like cells and germlings, IL-10 production was higher when the latter organism was used to stimulate human PBMCs. Since the cytokine profile stimulated by *S. schenckii sensu stricto* seems to be more pro-inflammatory than the one produced upon interaction with *S. brasiliensis*, it is tempting to speculate that this observation can be related to the severity of the lesions produced by these two species: *S. schenckii sensu stricto* usually generates limited lesions that can be spontaneously controlled by the infected subject; while *S. brasiliensis* often generates severe lesions, with low auto-resolution that usually leads to death (Castro et al., 2013; Mora-Montes et al., 2015). Furthermore, a lesser pro-inflammatory scenario stimulated by *S. brasiliensis* conidia and germlings could be translated in a better ability to colonize tissues and to establish the infection. Similarly, for the case of yeast-like cells this could help them to easily spread within the lymphatic system and deep organs. Nonetheless, additional experiments are required to support this hypothesis. It has been demonstrated in several species of *Candida* and *A. fumigatus* that exposure of inner wall components, by heat killing or exposing cells to sub-lethal concentrations of caspofungin, positively affects the stimulation of cytokine production, and this is mainly driven by recognition of β1,3-glucan via dectin-1 (Steele et al., 2005; Wheeler and Fink, 2006; Gow et al., 2007; Mora-Montes et al., 2010a; West et al., 2013; Estrada-Mata et al., 2015; Navarro-Arias et al., 2016; Perez-Garcia et al., 2016). Interestingly, here we did not find a similar result when either conidia or germlings from both *Sporothrix* species were used to stimulate cytokine production by human PBMCs. On the other hand, the use of laminarin to block dectin-1 indicates this receptor is a key element for cytokine production stimulated by the three morphologies of *S. schenckii sensu stricto*. In the case of *S. brasiliensis*, only yeast-like cells showed a similar trend, being dectin-1 required only to produce IL-10 stimulated by conidia, and dispensable for the cytokine production stimulated by germlings. This dataset indicates that dectin-1 is a relevant receptor for cytokine production during *S. schenckii sensu stricto*-human PBMC interaction and that most of the β1,3-glucan required to trigger dectin-1-dependent signaling pathways is already accessible at the cell surface. A similar scenario would apply for the case of *S. brasiliensis* yeast-like cells. Accordingly to our observation related to *S. brasiliensis* germlings, it has been reported that dectin-1 is dispensable to control *S. schenckii* in infected rats (Zhang et al., 2012).

The receptors TLR2 and TLR4 have been demonstrated to be relevant during the control of *S. schenckii sensu stricto* in a murine model of sporotrichosis (Sassá et al., 2012; Negrini et al., 2013). Here, we confirm these data using primary immune cells and demonstrated that they are also relevant during the interaction of *S. brasiliensis* with immune cells. Contrary to the previously published data, we found a lesser important role for TLR4 during human PBMCs interaction than that reported in the model of murine sporotrichosis; most probably due to the natural differences between the animal and human immune cells. The role of MR in the phagocytosis of different morphologies of *S. schenckii sensu stricto* was previously addressed, being more relevant for the uptake of yeast-like cells than conidia (Guzman-Beltran et al., 2012). Our result here indicate that this receptor has a minor contribution in the cytokine production stimulated by *S. schenckii sensu stricto* yeast-like cells and germlings, but conidia stimulated significant levels of pro-inflammatory cytokines via this receptor. Interestingly, yeast-like cells, but not other morphologies of *S. brasiliensis* required MR to stimulate pro-inflammatory cytokines.

## Conclusion

We reported here that two members of the *S. schenckii* complex, *S. brasiliensis* and *S. schenckii sensu stricto*, have similar cell wall composition, which undergoes changes depending on the cell morphology. The differences on the cell wall composition are likely to influence the contribution of immune receptors involved in the stimulation of cytokine production in human PBMCs.

## Author Contributions

JM-A and HM-M conceived the study; JM-A, LP-G, and EM-M performed the experiments; JM-A, LP-G, EM-M, ML, IM-D, LL-B, and HM-M analyzed the data; JM-A and HM-M drafted the paper; JM-A, LP-G, EM-M, ML, IM-D, LL-B, and HM-M approved the final version of the manuscript

## Conflict of Interest Statement

The authors declare that the research was conducted in the absence of any commercial or financial relationships that could be construed as a potential conflict of interest.
